# Role of tumour-associated macrophages in oral squamous cells carcinoma progression: an update on current knowledge

**DOI:** 10.1186/s13000-017-0623-6

**Published:** 2017-04-05

**Authors:** Maria Noel Marzano Rodrigues Petruzzi, Karen Cherubini, Fernanda Gonçalves Salum, Maria Antonia Zancanaro de Figueiredo

**Affiliations:** 1 Postgraduate Program in Dentistry, Pontifical Catholic University of Rio Grande do Sul (PUCRS), Porto Alegre, Brazil; 2 Hospital São Lucas da Pontifícia Universidade Católica do Rio Grande do Sul (PUCRS), Av. Ipiranga, 6690 – Ipiranga, Porto Alegre, RS CEP: 90610-000 Brazil

**Keywords:** Oral cancer, Oral squamous cell carcinoma, Head and neck cancer, Macrophage activation

## Abstract

**Background:**

Oral squamous cell carcinoma (OSCC) accounts over 90% of malignant neoplasms of the oral cavity. This pathological entity is associated to a high mortality rate that has remained unchanged over the past decades. Tumour-associated macrophages (TAMs) are believed to have potential involvement in OSCC progression. However, the molecular networks involved in communication between stroma and cancer cells have not yet been fully elucidated.

**Main body:**

The role of M2 polarized cells in oral carcinogenesis is supported by a correlation between TAMs accumulation into OSCC stroma and poor clinical outcome. Signalling pathways such as the NF-κB and cytokines released in the tumour microenvironment promote a bidirectional cross-talk between M2 and OSCC cells. These interactions consequently result in an increased proliferation of malignant cells and enhances aggressiveness, thus reducing patients’ survival time.

**Conclusions:**

Here, we present a comprehensive review of the role of interleukin (IL)-1, IL-4, IL-6, IL-8, IL-10 and the receptor tyrosine kinase Axl in macrophage polarization to an M2 phenotype and OSCC progression. Understanding the molecular basis of oral carcinogenesis and metastatic spread of OSCC would promote the development of targeted treatment contributing to a more favourable prognosis.

## Background

Cancer pathogenesis events take place in imbalanced microenvironments, where pathological states do not affect only neoplastic cells [1–3]. Instead, cancerous cells disrupt tissue homeostasis, disturbing different cell types and contributing to disease progression, through interactions with mediators of the immune system [4–6]. Oral squamous cell carcinoma (OSCC) is a solid tumour of epithelial origin that affects more than 400,000 individuals annually worldwide [7]. The mortality rate of this disease has remained largely unchanged for the last decades, with a 5-year survival under 50% [8]. There is compelling evidence that tumour-associated macrophages (TAMs) have potential involvement in the progression and metastatic spread of OSCC. The most important features associated with their presence in the lesion stroma include facilitation of angiogenesis, tumour cell invasion, augmentation of cell motility, persistent growth, and suppression of anti-tumour responses [9–13]. The signals involved in communication between tumour cells and macrophages have not yet been completely elucidated. However, the interaction among tumour and inflammatory cells seems to be bidirectional [14]. Here, we present the strategies by which tumour cells influence macrophage physiology to display a pro-tumour phenotype, and the contribution of TAMs to OSCC progression. Is given an overview of potential markers that could provide support for diagnostic, evaluation of clinical outcome, and be used as valuable antineoplastic targets.

### Macrophage differentiation

In adults, inflammatory monocytes (CD64^+^/CD16^-^ CCR2^+^ Ly6C^+^) constitutively originate tissue-resident macrophage populations [15, 16]. The exposure of these cells to microenvironmental stimuli results in complex phenotypic modifications in a time- and location-dependent manner [17–19]. The activation of different regulatory mechanisms and transcription pathways result in a vast spectrum of macrophage subtypes, of which M1 and M2 represent the extreme polarization phenotypes [18, 19]. The M1 polarization state depends on microbial stimulus and a T helper type 1 (T_H_1) cytokine profile (classical activation pathway). Whereas M2 polarization depends on a T helper type 2 (T_H_2) cytokine profile (alternative activation pathway) [20]. Interferon-gamma (INF-$\gamma$) and interleukin (IL)-4 secretion sustain an M1 and an M2 phenotype commitment, respectively [21]. M1 are innate immune effector cells that fight intracellular microbial challenges by means of reactive oxygen species and nitrogen intermediates. Activation of signal transducer and activator of transcription (STAT)-1 in M1 macrophages is important for optimal T_H_1 responses [22], such as direct tumour cell death [23, 24]. M2 macrophages block T_H_1 and differentiate in the tumour stroma from blood monocytes, or resident macrophages in resting state, after making contact with neoplastic cells presenting aberrant production of certain cytokines [18]. Additionally, they promote cancer progression by STAT-3 activation, inducing and maintaining a pro-carcinogenic inflammatory microenvironment [25].

### OSCC cells and TAM interactions

**Fig. 1:**
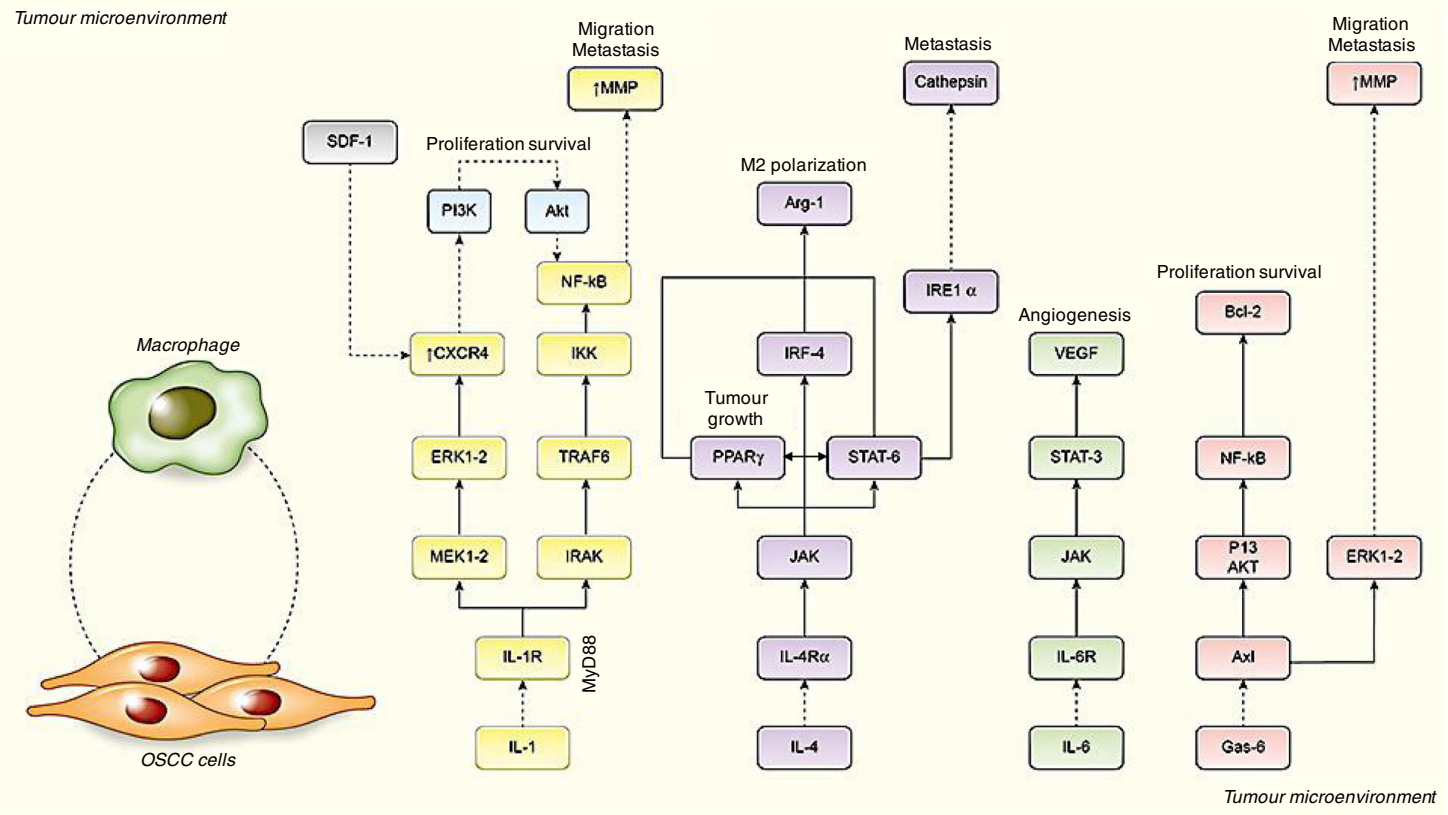
Macrophages in resting state suffer microenvironmental effects coordinated by OSCC cells. Interleukin (IL)-1, IL-4, IL-6, IL-8 and IL-10 (not shown), and Gas-6 are produced by OSCC cells and promote macrophage phenotype switching to an M2 polarization state. In turn, TAMs augment the recruitment of chemotactic receptors to tumour sites, induce tumour proliferation, and favour angiogenesis and invasiveness [31, 32, 36, 40–43, 47, 48, 65, 66]

Histopathologically, OSCC presents as fibrous connective tissue with unusual amounts of extracellular matrix rich in fibroblasts, vascular vessels, and inflammatory cells [26]. Among the local milieu of OSCC stromal spaces, rich in perlecans and inflammatory cells, monocytes or resting macrophages are differentiated into LyC16^high^, CD163^+^, CD204^+^, and CD68^+^ expressing TAMs. These cells are considered of utmost biological importance for disease progression and correlate with increased dedifferentiation in primary tumour sites [27–29]. Moreover, TAMs elicit tumour relapse and/or post-operative cervical lymph node metastasis via angiogenesis and suppression of anti-tumour immunity [9]. An increase in the number of CD163^+^ macrophages occurs in oral leukoplakia. However, they co-express phosphorylated STAT-1, suggesting that in premalignant lesions TAMs possess an M1 phenotype in a dominant T_H_1 microenvironment [30]. Polarization to an M2 TAM phenotype probably occurs gradually and early during the onset of cancer. It is suggested that several interleukins (IL-1, IL-4, IL-6, IL-8, and IL-10), and other factors, such as the receptor tyrosine kinase Axl, participate in promoting this phenomenon. In the next sections we propose a topic structured discussion of relevant findings that corroborate this theoretical assumption. Figure 1 briefly reviews the effect of interleukins on TAMs present in OSCC stroma.

### IL-1

Tumour released IL-1 cross-talks to TAMs and induces M2 polarization to an immunosuppressive phenotype via IL-1 receptor (IL-1R) and myeloid differentiation primary response gene 88 (MyD88), which requires I-kappaB kinase beta (IKKβ)-mediated nuclear factor kappa B (NF-κB) [31, 32]. Interleukin-1 beta (IL-1β) is a critical mediator of chronic inflammation and is implicated in OSCC during early and late stages of carcinogenesis. Pro-IL-1β is upregulated in tobacco and betel quid related oral cancer, and is secreted in an inflammasome-dependent manner [33], although it is absent in homeostatic conditions. In the presence of IL-1β TAMs suffer an upregulation of C-X-C motif chemokine receptors (CXCR), especially CXCR4, induced by the activation of extracellular signal regulated MAP kinase (ERK). Macrophages then become attracted by CXCR4 ligands, like stromal cell derived factor-1 alpha (SDF-1α) [34]. SDF-1α is highly inducible in hypoxic and proangiogenic niches, where it reinforces the autocrine/paracrine loop that contributes to an M2 phenotype [35]. Nevertheless, OSCC cells and TAMs together through IL-1β/IL-1R and CXCR4/ SDF-1α and via activation of the ERK signalling pathway produce tumour cell migration and invasion by inducing expression of matrix metalloproteinase (MMP) enzymes MMP-9 and MMP-13 [36]. These important angiogenic modulating enzymes promote the acquisition of vasculature for oxygenation, nutrition, and waste disposal, which are of fundamental importance for tumour growth [2]. Contrarily, the blockade of CXCR4 by the antagonist 1,1′-[1,4-phenylenebis(methylene)]bis-1,4,8,11-tetraazacyclotetradecane octahydrochloride (AMD3100) inhibited SDF-1 mediated lymph node metastasis [37]. Furthermore, a rich IL-1β microenvironment promotes CXCL1 production, and through CXCR2 this induces tyrosine phosphorylation of the endothelial growth factor receptor (EGFR) [33]. As a result, EGFR activates pathways leading to cell growth, DNA synthesis, and the expression of oncogenes like *fos* and *jun* [38]. IL-1α is found in tumour cell membranes and in intracellular locations, and is produced in larger amounts than IL-1β in highly metastatic tumours. Despite the lack of evidence that demonstrates its direct participation in OSCC progression through macrophage activation, IL-1α interacts with fibroblasts in the stroma. Therefore, IL-1α acts promoting cell proliferation and upregulating the secretion of IL-8, CXCL-1, and chemokine C-C motif ligand (CCL)-7 [39]. Coincidently, these cytokines are also commonly produced by TAMs, rising the hypothesis of a plausible interaction between OSCC and M2 cells by means of IL-1α.

### IL-4

IL-4 is an anti-inflammatory and immunomodulatory cytokine that has been identified as a relevant factor for the activation of TAMs, as well as IL-1. Furthermore, an increased expression of IL-4 receptor alpha (IL-4Rα) correlates with increased OSCC recurrence [40]. Regarding this tumour entity, the interaction between malignant cells and TAMs occurs through the plasminogen activator urokinase (uPA) and its specific receptor uPAR, mainly through the activation of ERK1/2 and increase in the production of IL-4. In OSCC cells this receptor modifies several transduction pathways, affecting neoplastic cell behaviours and acts as a promoter of survival, proliferation, and metastasis [41–43]. The high levels of IL-4 produced modifies the tumour microenvironment and facilitates an increase in arginase-1 levels, considered a biomarker of TAMs [43]. Similarly, this citokine induces cathepsin protease activity in TAMs, where they activate proteins including growth factors, transcription factors, and other proteases, such as MMPs [44]. Cathepsin B is considered a reliable marker for OSCC poor prognosis, correlating to higher tumour grade and lymph node metastasis [45].

### IL-6

IL-6 expression in OSCC has been related to high lymph node metastatic rates and poor tumour differentiation, especially in male patients [46]. SDF-1alpha increases secretion of IL-6 in cultured human OSCC cells via CXCR4, ERK, and NF-κB pathways [47], in a similar manner to that seen for IL-1β/IL-1R. Moreover, the aberrant synthesis of IL-6 by neoplastic cells may be controlled by the CXCR4-specific inhibitor AMD3100 [47]. The calcium binding protein S100A9, associated with loss of differentiation and recurrence, tends to be deregulated in both tumour and stromal cells. The expression of S100A9 in monocytes exerts a tumour-promoting effect upon co-culture with oral cancer cells, in particular by releasing IL-6 and the activation of NF-κB or STAT-3 that is not achieved in tumour cell monoculture [48]. In response to apoptotic tumour cell supernatants, signalling patterns were identified that contributed to the TAMs phenotype. Two targets, IL-4Rα and cannabinoid receptor 2 (CB2), were validated and confirmed to regulate both IL-6 and IL-10 production in TAMs, contributing to autocrine/paracrine activation of STAT-3 in macrophages and tumour cells [49]. These findings emphasise the relevance of tumour cells and TAMs interactions for disease progression.

### IL-8

IL-8 is a pro-angiogenic, pro-inflammatory mediator important for OSCC angiogenesis progression [50]. The mitogen activated protein kinases (MAPK) pathway is used by OSCC IL-8 to activate angiogenic activity in TAMs [51] augmenting, for example, vascular endothelial growth factor (VEGF) production. The receptors CXCR1 and CXCR2 have been detected in both oral normal keratinocytes and OSCC cells, where they exhibit higher expression. The presence of IL-8 CXC receptors in tumour cells increases ERK phosphorylation and MMP-7 and MMP-9 release, representing a tendency to proliferation, migration, and invasion [52]. Is important to consider that matrix metalloproteinase enzymes are essential for the achievement of a complete angiogenic potential of TAMs. At the same time, the progressive development of the tumour requires vast vasculature. Chronic periodontitis and tobacco consumption have both historically been associated to oral cancer. Then, recent published works propose the following interesting associations that also support the important role of IL-8 in OSCC progression. It is probable that *Porphyromonas gingivalis* contributes to OSCC progression, increasing IL-8 levels in the microenvironment and upregulating MMPs [53]. Nicotine also increases IL-8 release in OSCC, binding to the nicotine acetylcholine receptor (nAChR) and inducing calcium influx, that phosphorylates Ca(2+)/calmodulin-dependent kinase II (CaMK II) and NF-κB [54].

### IL-10

In more dedifferentiated tumour niches the microenvironment progressively acquires an immunosuppressive profile [1–4]. IL-10 is a cytokine that modulates immune responses, causing suppressive regulatory T cell differentiation that contributes to tumour cell proliferation [55]. Since persistent viral infection promotes IL-10 upregulation and impaired T-cell responses [56], it is believed that this cytokine plays a critical role in human papilloma virus (HPV)- and Epstein-Barr virus (EBV)-related OSCC progression [57, 58]. Moreover, IL-10 indicates poor outcomes in HPV-unrelated OSCC, especially when INF-γ secretion [59] and transforming growth factor beta 1 (TGF-β1) levels [60] are low. Receptors for IL-22, a member of the IL-10 family, are highly expressed in OSCC cells, including in metastatic sites, compared to healthy regions. It was observed that in the OSCC MISK81-5 cell line, IL-22 induced the translocation of phosphorylated STAT-3 and upregulated the expression of Bcl-xL, survivin, and c-Myc, all known anti-apoptotic genes, as well as suppressor of cytokine signalling 3 (SOCS3) [61]. In this context, diverse pathways for IL-10 production by TAMs have been described, highlighting their contribution to an immunosuppressive state in the tumour stroma. TAMs present a defective TLR response caused by tumour-selective disruption of the MyD88 signalling cascade, and affect the TIR-domain-containing adapter-inducing interferon-β (TRIF)/TNF receptor associated factors (TRAF3)-dependent pathway in their own favour, leading to favourable transcription at the IL-10 promoter region [62]. In the presence of apoptotic tumour cell-factors like sphingosine-1-phosphate (S1P), TAMs use tyrosine kinase receptor A (TRKA), phosphatidylinositol 3-kinase (PI3K)/protein kinase B (Akt), and MAPK signalling to induce IL-10 [63].

### Gas6/Axl

TAMs acquire, possibly by cancer-derived factors like IL-10, the capacity to produce high levels of Gas-6 that promotes tumour development [64]. At the same time, in a bidirectional interaction, OSCC cells, that also produce Gas-6, polarize TAM toward a tumour-promoter phenotype. In OSCC, Gas-6 cooperates with Axl and achieves biological and clinical relevance by triggering the signalling pathways of PI3/Akt and NF-κB [65]. TAMs and OSCC interact in Gas-6/Axl axis-modulated epithelial-mesenchymal transition by upregulating cadherin, n-cadherin, and vimentin expression, and promoting cell invasion and migration. It was found that Axl expression correlates with clinical stage and lymph node status in OSCC patients. Moreover, TAMs count was associated with phosphorylated Axl immunoactivity in OSCC tissues [13, 65, 66]. Gas-6/Axl and NF-κB may be interesting targets for therapeutic intervention, since NF-κB promotes cancer resistance to apoptosis and production of growth factors in the stroma, which stimulates tumour progression [31].

### Role of TAMs in OSCC histopathological diagnosis

Although further clinicopathological studies are needed before interactions between stromal cells and malignant cells can be defined as a key process for OSCC progression, evidence suggests that TAMs play several tumour-promoter roles during carcinogenesis [10, 28]. The presence of these polarized cells should be used as a potential marker to distinguish incipient OSCC from invasive lesions, avoiding underdiagnoses. As indicated by Matwaly et al. [67], the oral mucosa lacks an objective, standard-like structure that is found in other anatomical regions like the oesophagus, which makes the detection of invasiveness in oral cancer demanding. For a better understanding of TAMs in OSCC, more studies are necessary to define, by means of gene profiling, macrophage subpopulations with different tumour promoting abilities. A better indicator of the dynamic regulation of macrophage phenotype may be cellular cytokines, evaluated by means of tests conducted over multiple time points [67]. However, this methodology is time and cost demanding and probably unfeasible in clinical situations, especially in less developed countries where the prevalence of OSCC is higher. However, from the available evidence, it is possible to suggest that screening for TAM markers in oral biopsies certainly may contribute to accurate assessment of OSCC behaviour, being a valuable tool for the estimation of prognosis in cases related and unrelated to viral infection [57–60, 68].

### New diagnostic alternatives

Weber et al. [27] propose that even trauma from incisional biopsies might influence tumour biology leading to a worse prognosis and increased risk of developing lymph node metastases in OSCC patients. A wound-healing reaction consecutive to tissue trauma probably provides a microenvironmental stimulus that affects macrophage polarisation [69]. Until the present, diagnostic procedures and therapeutic planning for OSCC have been supported mainly by histopathological findings. Despite being inviable at present, mostly due to the lack of standardized techniques, interpretation, and validation of parameters, the development of new minimal invasive diagnostic strategies should consider the screening of salivary and serum markers that reflect tumour behaviour, associated or not with the improvement of classical techniques like exfoliative cytology. Several studies have demonstrated valuable associations among OSCC clinical stages and prognosis, and salivary or serum markers associated with TAM’s dynamic participation in the tumour stroma [58, 68, 70–73]. Although salivary markers associated to TAM polarization are not yet used as parameters for definitive diagnoses, they should be taken into consideration to evaluate patients with potent malignant disorders, like proliferative verrucous leukoplakia [74], as well as for recurrence in OSCC treated patients.

### Targeting TAMs in OSCC therapeutics

TAMs are potential targets for combination therapy in cancer treatment [75]. As we move forward, comprehension of the role of stromal cells in OSCC progression, suggest that therapies that only target TAMs may be possible, leading to an imbalance in tumour growth and invasiveness [75, 76]. However, despite its conceivable relevance, essentially mostly from positive clinical implications, the research in this field is incipient among cancer researchers. Recently a few studies have proposed targeting TAMs pathways to block cancer development [77, 78]. Signalling pathways such as the NF-κB and cytokines released in the tumour microenvironment through OSCC cells and TAMs interactions are attractive targets [79]. Inhibitors of cytokines involved in tumour signalling present potential for use to combat cancer, specially those implicated in promoting a malignancy cycle between OSCC cells and TAMs. Considering that chirurgical approaches are gold standard procedures for OSCC treatment, chemical interventions would be considered of lesser importance. However, it is relevant to underscore that during the last 30 years the disease-free survival and overall survival rates of OSCC patients have remained unchanged, perhaps due to limited care access or professional failures in performing early diagnoses, which is of the utmost relevance for prognosis. For these cases in particular, new therapeutic options are urgently needed.

## Conclusions

Impaired tumour-preventive responses in OSCC are promoted by malignant cells and by soluble factors of the microenvironment that attract and polarize macrophages to a tumour-promoting state. Besides, macrophages reinforce the loop that promotes cancer growth and metastasis. This link between inflammation and cancer regulate OSCC progression and signalling pathways that provide a cross-talk between cancer cells and TAMs should be taken into consideration as valuable antineoplastic targets.

## Abbreviations

Akt: Protein kinase B
AMD3100: 1,1′-[1,4-phenylenebis(methylene)]bis-1,4,8,11-tetraazacyclotetradecane octahydrochloride
Axl: Axl receptor tyrosine kinase
CaMK II: Ca(2+)/calmodulin-dependent kinase II
CB2: Cannabinoid receptor 2
CCL: Chemokine C-C motif ligand
CXCR: C-X-C motif chemokine receptors
EBV: Epstein-Barr virus
EGFR: Endothelial growth factor receptor
EMT: Epithelial-mesenchymal transition
ERK: Extracellular signal regulated MAP kinase
Gas-6: Growth arrest specific gene-6
GM-CSF: Granulocyte macrophage colony stimulating factor
HPV: Human papilloma virus
IKKβ: I-kappaB kinase beta
IL: Interleukin
IL-1R: Interleukin-1 receptor
INF-$\gamma$: Interferon-gamma
MAPK: Mitogen activated protein kinases
MMP: Matrix metalloproteinase
MyD88: Myeloid differentiation primary response gene 88
nAChR: Nicotine acetylcholine receptor
NF-κB: Nuclear factor kappa B
OSCC: Oral squamous cell carcinoma
PI3K: Phosphatidylinositol 3-kinase
S1P: Sphingosine-1-phosphate
SDF-1α: Stromal cell derived factor-1 alpha
SOCS3: Suppressor of cytokine signalling 3
STAT: Signal transducer and activator of transcription
TAMs: Tumour-associated macrophages
TGF-β1: Transforming growth factor beta 1
T_H_1: T helper 1
T_H_2: T helper 2
TLR: Toll-like receptors
TNF: Tumour necrosis factor
TRAF3: TNF receptor associated factors
TRIF: TIR-domain-containing adapter-inducing interferon-β
TRKA: Tyrosine kinase receptor A
uPA: Plasminogen activator urokinase
uPAR: Plasminogen activator urokinase receptor
VEGF: Vascular endothelial growth factor
